# Plasma exchange therapy for familial chylomicronemia syndrome in infant: A case report

**DOI:** 10.1097/MD.0000000000029689

**Published:** 2022-08-12

**Authors:** Lei Han, Guangfeng Qiang, Lei Yang, Rui Kou, Qiubo Li, Meiyun Xin, Ruihan Liu, Zhengjun Zhang

**Affiliations:** a Department of Pediatrics, Affiliated Hospital of Jining Medical University, Jining, China; b Department of Endocrinology, Affiliated Hospital of Jining Medical University, Jining, China.

**Keywords:** familial chylomicronemia syndrome, lipoprotein lipase, plasma exchange

## Abstract

**Introduction:**

Familial chylomicronemia syndrome (FCS) is a rare genetic disease. FCS usually manifests by the age of 10 years, and 25% of cases of FCS occur during infancy. Here we present a case of FCS in a male infant and summarize our experiences on the diagnosis and therapy of this case.

**Patient concerns:**

A male infant aged 1 month and 8 days had recurrent hematochezia and hyperchylomicronemia.

**Diagnosis:**

FCS based on symptoms and genetic test.

**Interventions:**

Plasma exchange therapy.

**Outcomes:**

His development was normal with a good spirit and satisfactory weight gain, and no hematochezia occurred again.

**Conclusion:**

Genetic test is important for accurate diagnosis of FCS, and we identified a new mutation of lipoprotein lipase gene c.88C>A which conformed to autosomal recessive inheritance. Plasma exchange therapy can be applied to infants with FCS with low risk and good outcomes.

## 1. Introduction

Familial chylomicronemia syndrome (FCS) is a rare genetic disease associated with elevated triglycerides and an increased risk of pancreatitis.^[1]^ FCS affects around 5000 patients globally and is characterized by severe hypertriglyceridemia, acute pancreatitis, recurrent abdominal pain, eruptive xanthomata, and lipemia retinalis. While the mutations in a series of genes have been identified to be implicated in the pathogenesis of FCS such as lipoprotein lipase (LPL), lipase maturation factor 1, apolipoprotein AV, apolipoprotein CII and glycosyl-phosphatidylinositol anchored high-density lipoprotein-binding protein 1, LPL mutations account for 80% cases of FCS.^[2]^ Therefore, FCS is a type of primary hyperlipoproteinemia and is also known as type I hyperlipoproteinemia.

Since the incidence of FCS is rare, published works of literature on FCS are limited. The diagnosis of FCS is mainly based on clinical symptoms, laboratory data, and genetic analysis.^[3]^ FCS usually manifests by the age of 10 years, and 25% of cases of FCS occur during infancy.^[4]^ Currently, the diagnosis and treatment of FCS is still a challenge, especially in infants. Here we present a case of FCS in a male infant and summarize our experiences on the diagnosis and therapy of this case.

## 2. Case report

This study was approved by the Ethics Committee of Affiliated Hospital of Jining Medical University. A male infant aged 1 month and 8 days had no history of hypoxic asphyxia at birth and was breastfed exclusively after birth. He was hospitalized in another hospital on October 03, 2018, due to hematochezia for 11 hours without obvious cause. His stool was mixed with blood streaks, and he had occasional crying during defecation, accompanied by more sleep. He had less milk consumption but had no fever, abdominal distension, or vomiting. He received an intramuscular injection of vitamin K1, but occult blood could still be seen in the stool reexamination. Therefore, he was admitted to our hospital.

Physical examination at admission showed the following results: body temperature 36.7°C, pulse 130 times/min, breathing 38 times/min, body weight 5.02 kg, the mind was clear, the stimulation response was poor, the complexion was red, and the breathing was smooth. His skin had no rash or nodular lesion. The anterior fontanel was flat and soft, and 3 concave sign was negative. Cardiopulmonary examination showed no abnormalities. The abdominal shape was normal and the liver and spleen had no swelling. The muscle tension of limbs was normal, the extremities were warm, and capillary refill time was less than 2 seconds.

Abdominal color Doppler ultrasonography showed a large amount of abdominal flatulence. The local intestinal structure of the left lower abdomen was slightly disordered, and necrotizing enterocolitis was indicated. Ultrasonographic examination of the abdominal aorta, bilateral neck arteries, and veins showed no abnormality. Color Doppler ultrasonography of the liver, gallbladder, pancreas spleen, and kidneys showed no abnormality. The results of routine stool tests and occult blood tests were weakly positive. procalcitonin level was 0.18 ng/mL, and urine amylase had no abnormality. Blood gas analysis showed the following results: pH 7.38, PCO2 33.1 mmHg, PO2 84.4 mmHg, BE-5.5 mmol/L, lactic acid 5.8 mmol/L, blood glucose 8.1 mmol/L. The blood biochemistry test showed the following results: K^+^ 5.23 mmol/L, Na^+^ 130 mmol/L, Cl^−^ 103 mmol/L, HCO3^−^<5.0 mmol/L. Blood routine test showed the following results: white blood cells 18.22 × 10^9^/L, red blood cells 1.97 × 10^12^/L, hemoglobin 130 g/L, platelets 361 × 10^9^/L, lymphocyte percentage 40.0%, monocytes 11.0%, neutrophils 45.0%. When a blood sample was taken from the infant, his blood was pink serous, and the upper layer showed milky white cream, indicating severe hyperlipidemia, but could not be detected by liver function, kidney function, myocardial enzyme, blood lipid, blood amylase, coagulation routine, D-dimer, and other laboratory tests.

After admission, the infant was given treatments such as antibiotics ceftriaxone sodium, fasting, and fluid replacement. He still had recurrent hematochezia and received plasma exchange therapy on the second day of admission. A total of 532.8 mL of type O RH positive red blood cells and 266.4 mL of homologous virus-inactivated plasma were transfused, and the total exchange blood was 799.2 mL (160 mL/kg). Intraoperative monitoring showed that arterial blood pressure fluctuated at 62–80/42–60 mmHg, and the urine volume was 174 mL. After plasma exchange therapy, the blood color was darker and the white cream-like component was significantly reduced. The blood test showed the following results: triglyceride (TG) > 50 mmol/L (normal value 0.56–1.7 mmol/L), total cholesterol 12.96 mmol/L (normal value 2.8–5.85 mmol/L), high-density lipoprotein cholesterol 0.21 mmol/L (normal value 0.8–2.0 mmol/L), low-density lipoprotein cholesterol 3.68 mmol/L (normal value 2.0–3.36 mmol/L), very-low-density lipoprotein cholesterol 9.07 mmol/L (normal value 3.13–3.59 mmol/L), lipoprotein a 1,225 mg/L (normal value 0–300 mmol/L). The test of organic acids in urine showed ketonuria and no other abnormalities. The test of organic acids in blood showed increased levels of carnitines and no other abnormalities.

After treatment, the blood in the stool of the infant significantly reduced, and he was fed with medium-chain fatty acid milk powder. His parents refused the second plasma exchange therapy and he was discharged after being hospitalized for 4 days. One week after discharge, the child was followed up. He was fed with medium-chain fatty acid milk powder at home, his hematochezia stopped 5 days after discharge, and there were no abdominal distension, vomiting or other symptoms. He visited the outpatient clinic of our hospital for reexamination on December 20, 2018 (74 days after discharge). His blood sample was bright red, blood triglyceride was 52 mmol/L, total cholesterol was 10.50 mmol/L, high-density lipoprotein cholesterol was 0.21 mmol/L, low-density lipoprotein cholesterol was 3.00 mmol/l, very low-density lipoprotein cholesterol was 7.29 mmol/L, lipoprotein a was 1,045 mg/L. In February 2019 (4 months after discharge), the child was followed up again. His development was normal with good spirit and satisfactory weight gain, and no hematochezia occurred again.

**Figure 1.**
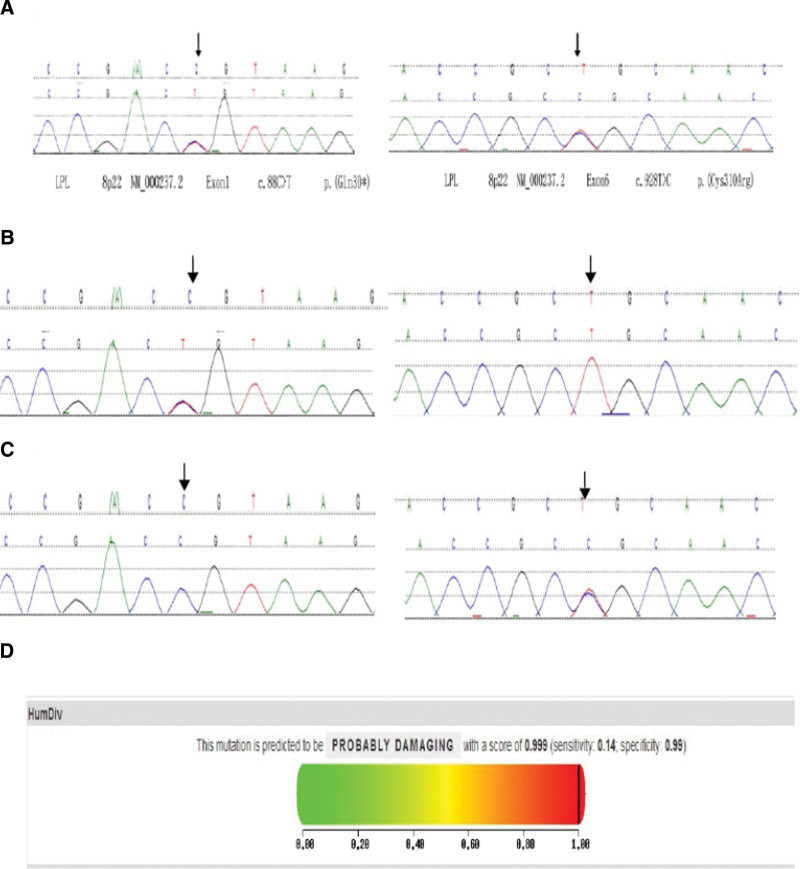
Gene sequencing results of the child and his parents. (A) Compound heterozygous mutation c.88C>T/c.928 T>C was detected in LPL gene in the child. (B) The father had nonsense mutation c.88C>A in exon 1, and exon 6 was normal. (C) The mother had missense mutation c.928 T>C in exon 6, and exon 1 was normal. (D) PolyPhen-2 software predicted that the mutation was “very likely to be harmful”, and the score was 0.999 (sensitivity 0.14, specificity 0.99). LPL = lipoprotein lipase.

After obtaining the informed consent of the family members, we performed a hyperlipidemia-related genetic examination. In LPL gene mutation analysis, the compound heterozygous mutation c.88C>T/c.928 T>C of exon 1 and exon 6 was detected in the infant (Fig. 1A). His father and mother were heterozygotes, the father carried the nonsense mutation c.88C>A in exon 1, and exon 6 was normal (Fig. 1B); the mother carried the missense mutation c.928 T>C in exon 6, and exon 1 was normal (Fig. 1C). The nonsense mutation c.88C>A in exon 1 changed the coding amino acid from glutamine to termination codon, and the missense mutation c.928 T>C in exon 6 changed the coding amino acid from cysteine to arginine. The prediction by PolyPhen-2 software suggested that the mutation was “very likely to be harmful” (Fig. 1D), and the score was 0.999 (sensitivity 0.14, specificity 0.99). The substitution of cysteine at p.310 with arginine is harmful, confirming that the mutation is a new pathogenic mutation.

## 3. Discussion

FCS mostly occurs before the patients are 10 years old, and about 25% of cases occur as early as infancy with blood samples appearing milk-like.^[4]^ The plasma TG level is usually above 11.3 mmol/L and abdominal pain and/or pancreatitis are often accompanied. About 50% of the patients develop eruptive xanthoma, especially at the compression site and extensor surface, and plasma TG level exceeds 22.6 mmol/L. FCS can also manifest as fatty retinitis and hepatosplenomegaly, and high-fat diet can aggravate symptoms by accumulating chylomicrons in the blood.^[5]^ Children with FCS can manifest pancreatitis, lipid encephalopathy, multiple organ damage, and fatal cases.^[6–9]^ Gastrointestinal hemorrhage caused by hyperlipidemia is very rare, high concentration of chyle may cause an increase in blood viscosity and intestinal vascular damage, resulting in necrosis and gastrointestinal hemorrhage.^[10]^ Therefore, the diagnosis and treatment of FCS are urgent.

In this case, we detected the LPL gene of the probands and their family members. Gene mutation analysis showed that the nonsense mutation c.88C>A in exon 1 would shorten the amino acid sequence and result in a change in the protein structure. We searched the literature and C. 88C > A has not been reported yet, indicating that we identified a new mutation. Another mutation is the missense mutation c.928 T>C in exon 6, which changes the amino acid from cysteine to arginine, and the prediction by PolyPhen-2 software suggested that the mutation is “very likely to be harmful”. His father is a heterozygote who carries the nonsense mutation c.88C>A in exon 1, and his mother is a heterozygote who carries the missense mutation c.928 T>C in exon 6. The parents had no clinical symptoms, consistent with Mendel’s law of inheritance and autosomal recessive inheritance.

At present, there is no specific drug for the treatment of FCS, and FCS is mainly managed by dietary control and plasma exchange.^[11,12]^ Plasma exchange is often the preferred method for FCS treatment, but it is difficult for newborns or infants under 3 months for the following reasons: (1) Plasma exchange requires deep venous puncture catheterization, while the punctures of femoral vein, internal jugular vein and subclavian vein in infants are all difficult and risky. (2) Plasma exchange of infants requires blood purifiers, filters, and pipelines, which are expensive and not easily available in most hospitals.^[13]^ Pugni et al. used exchange transfusion for a 23-day-old patient with hypertriglycemia, and TG level was significantly and rapidly decreased, and then was maintained in a normal range by dietary control.^[14]^ Volanesorsen is a promising ApoIII inhibitor and has been recently applied to improve lipid profiles in patients with diabetic dyslipidemia.^[15]^ Further studies are needed to examine whether volanesorsen could be used to improve lipid profiles in FCS patients In this case, because the child had severe hypertriglyceridaemia and TG was greater than 50 mmol/L, we chose synchronous plasma exchange of peripheral artery and vein. After treatment, the condition of the child improved and no severe complications occurred.

In summary, we made an accurate diagnosis of FCS based on symptoms and genetic tests, and identified a new mutation of LPL gene C. 88C>A which conformed to autosomal recessive inheritance. In addition, we successfully applied plasma exchange therapy for an infant with FCS with low risk and good outcomes.
